# The role of eye-specific attention in ocular dominance plasticity

**DOI:** 10.1093/cercor/bhac116

**Published:** 2022-03-24

**Authors:** Fangxing Song, Lili Lyu, Jiaxu Zhao, Min Bao

**Affiliations:** CAS Key Laboratory of Behavioral Science, Institute of Psychology, Chinese Academy of Sciences, 16 Lincui Road, Chaoyang District, Beijing 100101, China; Department of Psychology, University of Chinese Academy of Sciences, 19 Yuquan Road, Shijingshan District, Beijing 100049, China; Key Laboratory of Primate Neurobiology, Institute of Neuroscience, CAS Center for Excellence in Brain Science and Intelligence Technology, Chinese Academy of Sciences, 320 Yue Yang Road, Shanghai 200031, China; CAS Key Laboratory of Behavioral Science, Institute of Psychology, Chinese Academy of Sciences, 16 Lincui Road, Chaoyang District, Beijing 100101, China; Department of Psychology, University of Chinese Academy of Sciences, 19 Yuquan Road, Shijingshan District, Beijing 100049, China; CAS Key Laboratory of Behavioral Science, Institute of Psychology, Chinese Academy of Sciences, 16 Lincui Road, Chaoyang District, Beijing 100101, China; Department of Psychology, University of Chinese Academy of Sciences, 19 Yuquan Road, Shijingshan District, Beijing 100049, China

**Keywords:** adaptation, attention, ocular dominance, ocular opponency neuron, SSVEP

## Abstract

It is well known how selective attention biases information processing in real time, but few work investigates the aftereffects of prolonged attention, let alone the underlying neural mechanisms. To examine perceptual aftereffect after prolonged attention to a monocular pathway, movie images played normally were presented to normal adult’s one eye (attended eye), while movie images of the same episode but played backwards were presented to the opposite eye (unattended eye). One hour of watching this dichoptic movie caused a shift of perceptual ocular dominance towards the unattended eye. Interestingly, the aftereffect positively correlated with the advantage of neural activity for the attended-eye over unattended-eye signals at the frontal electrodes measured with steady-state visual evoked potentials. Moreover, the aftereffect disappeared when interocular competition was minimized during adaptation. These results suggest that top-down eye-specific attention can induce ocular dominance plasticity through binocular rivalry mechanisms. The present study opens the route to explain at least part of short-term ocular dominance plasticity with the ocular-opponency-neuron model, which may be an interesting complement to the homeostatic compensation theory.

## Introduction

Our brain can selectively process a subset of input that is most relevant to behavioral goals. This confined sensory processing is known as selective attention (Treisman 1969; Wolfe et al. 1989). A pioneer study on selective attention introduces a selective-looking paradigm (Neisser and Becklen 1975), where subjects were shown 2 disparate episodes in spatial overlap or 1 episode in each eye (i.e. dichoptic display). It was found that subjects could follow the action in one episode and ignore the other and rarely noticed odd events in the unattended episode, showing how powerful selective attention filtered input information.

To date, it is known that selective attention can bias information processing by enhancing processing of attended stimulus (Corbetta et al. 1990; Motter 1994; Mangun 1995) and suppressing processing of unattended stimulus (Rees et al. 1997; Vidnyánszky and Sohn 2005; He et al. 2021). Despite numerous research on real-time effects of selective attention, much fewer work has measured perceptual aftereffects of prolonged selective attention (Wang et al. 2021), and to our best knowledge, no one has explored its underlying neural mechanism. Here we tested perceptual consequence after prolonged attention to a monocular pathway using both psychophysical and steady-state visual evoked potential (SSVEP) measurements.

**Fig. 1:**
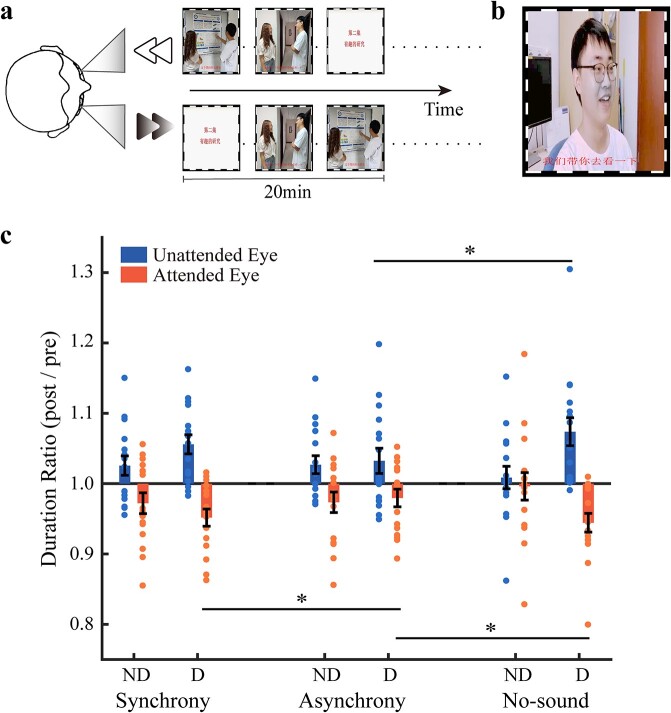
Illustration of the “dichoptic-backward-movie” paradigm (a), the blob target (see the gray region around the mouth in this example) in experiment 1b (b), and the mean duration ratios for the 3 adaptation conditions in experiment 1a (c). The circles show the individual data. Error bars represent standard errors of means. Asterisks represent significant differences between different adaptation conditions (FDR correction, ^*^P < 0.05). For demonstration purpose only, the images with human faces shown in this figure are photographs taken from 2 graduates of our lab who gave permission to publish their identifiable images. The movie images we actually used in the experiment are not shown here due to copyright concerns.

For this goal, we developed a “dichoptic-backward-movie” adaptation paradigm enlightened by the selective-looking paradigm (Neisser and Becklen 1975). During adaptation, movie images played normally were presented to one eye, while movie images of the same episode but played backwards were presented to the other eye (Fig. 1a). In this paradigm, the overall interocular contrast energy was balanced throughout the adaptation period. Thus, any change of ocular dominance after adaptation cannot be simply ascribed to unbalanced input energy as in monocular deprivation (Lunghi et al. 2011; Zhou et al. 2014; Bai et al. 2017).

Note that the movie plot of backward movie images was illogical and hard to follow. Subjects were thus instructed that their primary task was to try their best to follow the logic of the regular movie and ignore the superimposed backward movie. Therefore, we believe that goal-directed selective attention was predominantly focused on the regular movie. Here the eye to which the regular movie images were presented was called the “attended eye,” and the opposite eye was called the “unattended eye.”

As voluntary attention can increase the proportion of time of perceiving inputs to the attended eye in binocular rivalry (Chong et al. 2005; Zhang et al. 2012; Marx and Einhauser 2015), we hypothesized that prolonged top-down eye-specific attention by watching the dichoptic movie would increase the probability of perceiving the regular movie images and boost the neural responses to signals from the attended eye. This would cause sustained unbalanced responses in the 2 monocular pathways to some degree resembling the neural activity pattern during short-term monocular deprivation (Lunghi et al. 2015; Lyu et al. 2020). We surmised that prolonged watching the dichoptic movie might also cause a shift of perceptual ocular dominance, as often seen in monocular deprivation studies (for review, see Basgoze et al. 2018; Bao and Engel 2019).

To test this hypothesis, in experiment 1, we measured perceptual ocular dominance before and after 1 h of watching the dichoptic movie by using a binocular rivalry task, which had been frequently adopted in monocular deprivation studies (Lunghi et al. 2013; Bai et al. 2017; Han et al. 2020; Lyu et al. 2020; Steinwurzel et al. 2020; Wang et al. 2021). Experiment 2 measured the SSVEP signals for each eye when subjects were watching the dichoptic movie, providing further neural evidence supporting our hypothesis. By alternating monocular presentations of the regular and backward movie images in the 2 eyes, binocular rivalry was largely avoided during the adaptation period in Experiment 3. Thus, experiment 3 further examined to what extent our findings in the first 2 experiments were related to the mechanisms of binocular rivalry.

## Materials and methods

### Experiment 1

#### Subjects

Twenty-two adult humans (13 females, 9 males; age range 18–25 years) participated in experiment 1a. Sixteen subjects (12 females, 4 males; age range 18–33 years) participated in experiment 1b. In all the experiments, all subjects had normal or corrected-to-normal vision, were unaware of the experimental hypotheses, and gave informed consent. All experimental procedures were approved by the Institutional Review Board of the Institute of Psychology, Chinese Academy of Sciences. The numbers of subjects were predetermined based on the sample sizes for published studies in this field.

#### Apparatus

The visual stimuli were programmed in MATLAB using the Psychtoolbox extensions (Brainard 1997; Pelli 1997). For 8 subjects of the “Synchrony” condition, stimuli were displayed on a gamma-corrected 21-inch Dell P1230 CRT monitor (at the refresh rate of 85 Hz and the mean luminance of 40.5 cd/m^2^). While for other subjects and other conditions, a gamma-corrected 21-inch Sun GDM 5510 CRT monitor (at the refresh rate of 75 Hz and the mean luminance of 42.8 cd/m^2^) was used. The spatial resolution of both monitors was 1,024 × 768 pixels. Subjects viewed the stimuli through a mirror stereoscope from a distance of 70 cm, with their heads stabilized in a chinrest. Experiments were conducted in a dimly lit room.

#### Stimuli and procedure of experiment 1a

##### Binocular rivalry test

Binocular rivalry stimuli were composed of 2 orthogonal sine-wave grating disks, oriented ±45° (diameter: 1°, spatial frequency: 3 cpd, Michaelson contrast: 80%). They were presented dichoptically and foveally with a central red fixation point (0.07° in diameter) and a high contrast checkerboard “frame” (size: 2.5° × 2.5°; 0.25° thick) to facilitate stable binocular fusion.

Each binocular rivalry test was composed of sixteen 60-s rivalry trials. Each trial started with a 5-s blank interval. Then, the rival stimuli were presented for 55 s. Subjects were required to hold down 1 of the 3 keys (left, right, or down arrow) to report their perceptions (clockwise, counterclockwise, or mixed). The orientation related to each eye was kept constant within a trial but randomly varied across the trials.

##### Dichoptic-backward-movie adaptation

During 1 h of adaptation, subjects passively viewed dichoptically presented movie images (Fig. 1a) surrounded by a high contrast checkerboard “frame” (size: 12.28° × 19.12°; 0.29° thick). The frame rate of movies was 25 fps. The original movie images were presented to 1 of the 2 eyes, while the corresponding backward movie images were presented to the opposite eye. The backward movie images were offline processed with 3 steps: dividing the regular movie into a series of 20-min segments, generating a backward copy for each 20-min segment in the MediaEditor software (http://www.aijianji.com/medownload.htm), concatenating the 3 segments to produce a backward movie file. That is, for each 20-min segment, the backward version of the movie was formally identical to the original one except for the absence of a logical movie plot.

For the first 6 subjects, the audio track always synchronized with the regular movie images (i.e. the “Synchrony” condition). To examine the potential contribution of audiovisual integration, we added another 2 different adaptation conditions for the 16 later recruited subjects. In the “Asynchrony” condition, the audio track was 5 s ahead of the movie image. Since the time window of audiovisual integration is only several hundred milliseconds wide (Conrey and Pisoni 2006), 5 s is sufficiently long to avoid audiovisual integration. In the “No-sound” condition, the movie was played silently.

##### Binocular rivalry practice

Before the formal experiment, all subjects practiced 3 binocular rivalry tests per day (with a 10-min break in between) for 3–7 days, to ensure a stable performance of binocular rivalry (Bao et al. 2018). Because perceptual eye dominance fluctuated widely in the first several trials of a day (Suzuki and Grabowecky 2007), before the practice of each day, subjects completed 5 warm-up binocular rivalry trials, the data of which were not analyzed.

##### Experimental design

Perceptual eye dominance was determined by the last 3 sessions of the “binocular rivalry practice” stage, with the dominant eye being the one that showed the longer summed phase durations.

The formal experiment included 4 phases: (1) 5 warm-up binocular rivalry trials (data not analyzed), (2) a preadaptation binocular rivalry test, (3) 1 h of dichoptic-backward-movie adaptation, and (4) a post-adaptation binocular rivalry test.

Subjects completed 4 sessions for each adaptation condition, with each eye attended in 2 sessions and the sequence counterbalanced. This resulted in totally 12 adaptation sessions. The order of the 3 adaptation conditions was balanced across subjects.

#### Analyses

To quantify the perceptual dominance of each eye, we calculated the summed phase durations of the exclusively monocular percepts and the mixed percepts across all the trials, respectively. Then, we computed the “duration ratio” for each eye using the formula (*T*_Post_ + *T*_Mpost_/2)/(*T*_Pre_ + *T*_Mpre_/2), where *T*_Post_ and *T*_Pre_ represented the summed phase durations of the exclusive percept for one eye during the post- and preadaptation tests and *T*_Mpost_ and *T*_Mpre_ represented the summed phase durations of the mixed percepts during the post- and preadaptation tests. If perceptual eye dominance shifts towards one eye after adaptation, the duration ratio would be greater than 1, and vice versa.

#### Stimuli and procedure of experiment 1b

The stimuli and procedures resembled those in experiment 1a except we added a blob detection task. Besides the primary task of watching regular movies, subjects had to detect a color desaturation within a blob region that was presented only to one eye. The center of the blob was completely desaturated (see Fig. 1b), while the color away from the center gradually restored in a 2D Gaussian profile (standard deviation 2.23° × 3.33°). According to our pilot experiment, subject usually paid attention to the faces of movie characters during adaptation. Therefore, a target presented within a face region would be detected much more likely than that presented elsewhere, which was in accord with the phenomenon of inattentional blindness (Jensen et al. 2011). To control this unwanted confounding factor, we predetermined the locations of the blob targets to ensure that every blob would appear on a face in the movie. Given this manipulation, we actually instructed subjects to press the SPACE bar as soon as they detected any part of a character’s face to turn gray.

Blobs were rare (once per 5 min in each eye) to ensure that watching movies was still the primary task. To determine the timing of the blobs, we divided the total adaptation period (60 min) into 24 segments (each 150 s long). Twelve of them were randomly assigned to each eye. A blob was presented at a random time within the intermediate interval (50 s long) of a segment, which would fade in within 0.2 s and after 5 s fade out also within 0.2 s. Before and after adaptation, binocular rivalry tests were not conducted in this experiment.

### Experiment 2

#### Subjects

Thirty-four volunteers received a screening test first (see Experimental Design below). Among them, 22 (8 males and 14 females, age range 19–28 years) passed the screening and then completed the formal experiment.

#### Apparatus

In the EEG experiment, Stimuli were presented on a gamma-corrected 21.5-inch LEN LS2224A LCD monitor at a resolution of 1,024 × 768 pixels and a refresh rate of 60 Hz. The mean luminance of the display was 43.9 cd/m^2^. Other apparatuses used in the experiment were the same as in experiment 1.

#### Stimuli and procedure

**Fig. 2:**
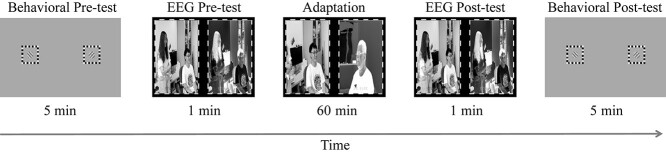
Diagram of the experimental session in experiment 2. A binocular rivalry pre-test preceded a preadaptation SSVEP test. After 60 min of adaption, a post-adaptation SSVEP test was completed followed by a binocular rivalry post-test. For the control session, the only difference was the adaptation phase, where both eyes viewed identical regular movie images though still flickering at the frequencies specified in the experimental session.

We used the frequency tag technique to obtain the SSVEP signal for each eye throughout the experiment (including the EEG pre-test, adaptation, and EEG post-test phases). Movie images in each eye were contrast-reversed flickering. The flickering frequency was 3 Hz for the unattended eye and 3.75 Hz for the attended eye. Each subject completed an experimental session and a control session (see Experimental Design below). During the adaptation phase (the 60-min phase in Fig. 2) of the experimental session, the regular movie images flickered in the attended eye; whereas the backward movie images flickered in the unattended eye. Yet during the adaptation phase of the control session, both eyes viewed identical regular movie images that flickered in the same way as in the experimental session. The control session evaluated the potential influence of flickering frequency on the SSVEP amplitude, which could be considered in the data analysis for the experimental session.

Before and after the adaption phase (see the 1-min phases in Fig. 2), both eyes were presented with identical regular movie images flickering for 1 min (Lyu et al. 2020). We used the same movie clips in the EEG pre- and post-tests to ensure that any difference of EEG signals was not caused by the movie content. Other stimuli and procedures (e.g. for the binocular rivalry and blob detection tasks) were as described in experiments 1a and 1b.

#### Experimental design

All subjects first practiced the binocular rivalry task. The performances of 5 subjects were still unstable after several days of practice, and 2 subjects were reluctant to complete the practice. Therefore, these 7 subjects quit the experiment.

After the practice stage, subjects were asked to complete a screen test in which they watched the flickering dichoptic movie; meanwhile, they performed the blob detection task. The aim was to screen subjects for their abilities to allocate eye-specific attention (Neisser and Becklen 1975). Only those who showed better blob detection performances in the attended eyes were eligible to start the EEG formal experiment. Four subjects failed to pass the screen test, and one withdrew from the experiment due to sickness.

In the EEG formal experiment, subjects first performed 2 binocular rivalry tests (Fig. 2). Each included 5 trials. The first 5 trials served as a warm-up test which were not analyzed (Bai et al. 2017; Bao et al. 2018). The second 5 trials were the binocular rivalry pre-test measuring the perceptual ocular dominance before adaptation. Subjects then performed an EEG pre-test measuring the neural ocular dominance when they watched natural scene stimuli before adaptation. This was followed by a 1-h dichoptic-backward-movie adaption. During adaptation, the primary task was still to follow the logic of the regular movie. The dichoptic movie was played with sound (synchronized with the regular movie) for ease of eye-specific attentional allocation. Meanwhile, subjects had to detect infrequent blob targets as in experiment 1b. To prevent fatigue, the 1-h adaptation was divided into two 30-min sections. Subjects were allowed to rest for 5 min with their eyes closed between the 2 sections. At the end of adaptation, subjects performed an EEG post-test followed by a binocular rivalry post-test.

Based on the findings of experiment 1a, the regular movie images were always presented to the dominant eye in this experiment (i.e. the attended eye was the dominant eye) to maximize the shift of ocular dominance. To avoid the potential frequency-dependent variances (for details see Section 3.3.3), each subject completed a control session in addition to the experimental session. The experimental and control sessions were conducted at least 24 h apart, with the order counterbalanced across subjects.

#### E‌EG data acquisition

EEG data were collected in a dark and confined laboratory using the Neuroscan Synamps2 system. Whole-brain signals were recorded by means of 64-channel (Ag-AgCl) electrode caps placed according to the International 10-20 system. The EEG signals were filtered from 0.05 to 100 Hz and an additional notch filter at 50 Hz and digitized at a sampling rate of 1,000 Hz. Impedances were kept below 5 kΩ. All electrodes were referenced online to the left mastoid (M1) electrode. Vertical electrooculogram was recorded by 2 electrodes placed above and below the left eye, with horizontal electrooculogram beyond the lateral canthi of both eyes.

#### Data analysis

##### Binocular rivalry task

We calculated the eye-ratio index for the pre- and post-test using the formula $({T}_{UAE}+{T}_M/2)/({T}_{AE}+{T}_M/2)$, where *T_UAE_*, *T_AE_*, and *T_M_* represented the summed phase durations for perceiving the stimulus in the unattended eye, stimulus in the attended eye, and mixed percepts, respectively.

##### E‌EG preprocessing

Off-line analysis was conducted using customized MATLAB codes and FieldTrip (Oostenveld et al. 2011). The M1 electrode was first removed from the raw data because of excessive noise. The EEG data were resampled to 1,024 Hz and digitally band-pass filtered from 1 Hz to 30 Hz, followed by an average reference. Then, a surface Laplacian spatial filter was used to minimize common noise (Hjorth 1975), subtracting the mean response of the nearest 4–8 electrodes from the signal of the central electrode. The EOGs and M2 were ignored during the surface Laplacian spatial filtering. Signals from the remaining electrodes were used to calculate the signal-to-noise ratio (SNR) and extract the amplitude of the frequency-tagged signal.

##### Extraction of SSVEP signals

Fast Fourier transform was applied to extract the power spectra. The SNR was computed as the ratio of the power at the tagged frequency (6 Hz and 7.5 Hz, i.e. the second harmonic of the flickering) to the average power of the 20 surrounding (10 on each side, excluding the immediately adjacent bin) frequency bins (Lyu et al. 2020). We used an adaptive recursive least square filter (Tang and Norcia 1995) to calculate the amplitude at the tagged frequencies using a 1-s sliding window (Zhang et al. 2011). The first 2 s of amplitude data were excluded to avoid the start-up transient. The remaining timecourse was averaged to compute the amplitude.

##### Selection of electrodes of interest

**Fig. 3:**
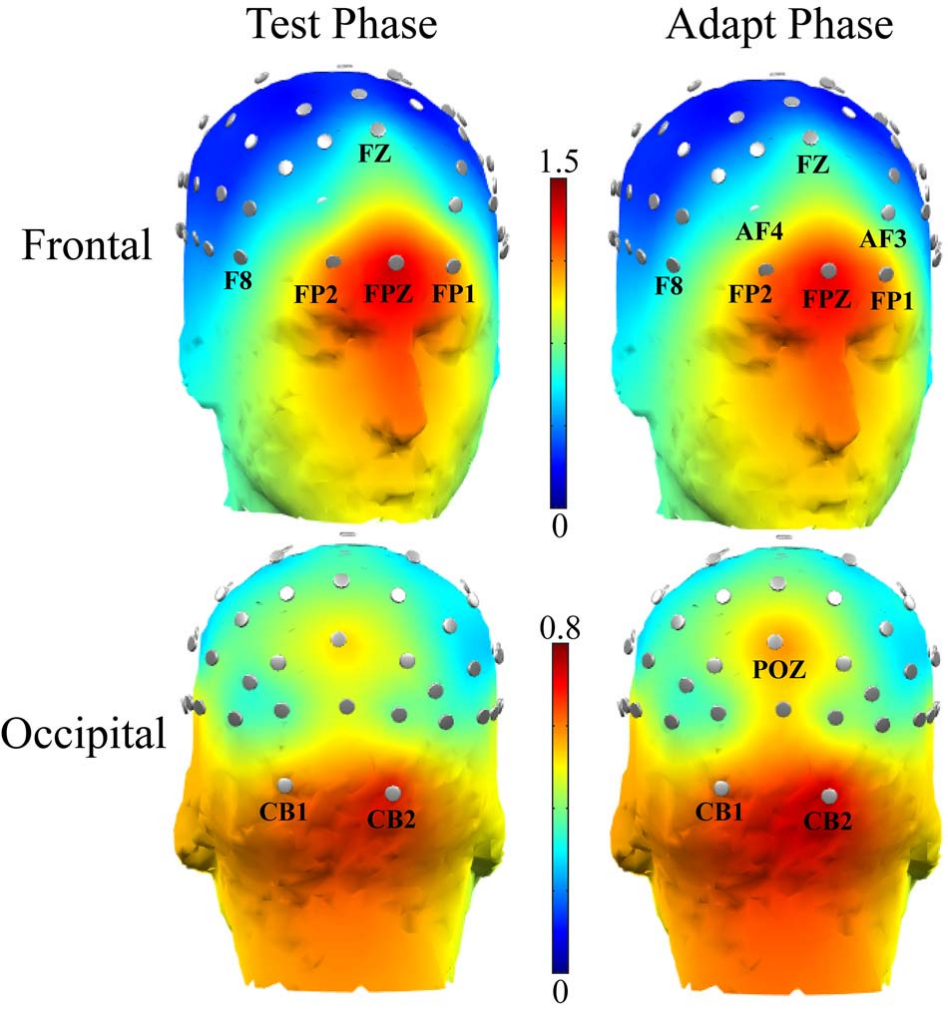
Average topography for the test phase and adaptation phase. The electrodes indicated in the figure are the selected EOIs.

To focus on the electrodes with sufficiently strong responses, for each electrode we compared the average SSVEP amplitude for both eyes in the pre- and post-tests with the grand mean amplitude across all the electrodes and subjects by using a one-sample *t*-test (Huang et al. 2018; Dong et al. 2020; Lyu et al. 2020). Only electrodes on which the amplitude was significantly beyond the grand mean were selected as electrodes of interest (EOIs; Fig. 3, see the Supplementary Material for details). Then, the SSVEP amplitudes of EOIs were averaged for statistical comparisons.

### Experiment 3

#### Subjects

Twenty volunteers received a screening test. Among them, 16 subjects (4 males and 12 females, age range 18–27 years) passed the screening and completed the formal experiment.

#### Stimuli and procedure

**Fig. 4:**
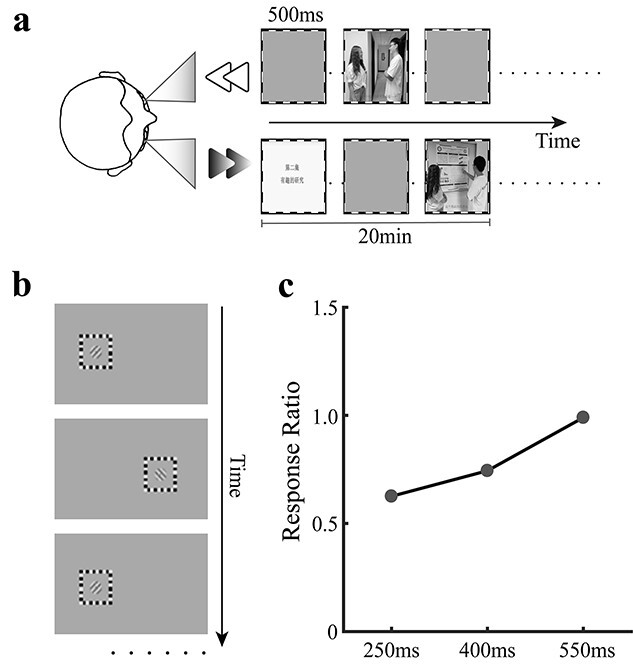
Diagrams for (a) the alternating presentation of dichoptic movie in experiment 3 and (b) the interval testing tasks. Typical results of the interval test for one participant (c). The response ratio was calculated by dividing the switch rate of responses by the alternation rate of grating presentations. A response ratio of 1 means that subject did not experience binocular rivalry at that interval. Response ratios smaller than 1 indicate the existence of binocular rivalry.

To minimize binocular rivalry as much as possible during adaptation, we dichoptically presented the regular and backward movie images to the 2 eyes in alternation (Fig. 4a). Thus, at any time, only one eye was stimulated, while the other eye viewed a midgray background. Meanwhile, we wanted the interval of each monocular presentation to be minimized, so that subjects did not have troubles in following the logic.

For this goal, each subject first conducted an interval test for estimating the shortest appropriate interval (Fig. 4b). The interval testing task resembled the binocular rivalry task, except that the grating in each eye was also presented in alternation for a specified duration. The subjects’ task was to track the grating orientation by pressing and holding the corresponding arrow key. If the interval of each monocular presentation was long enough, no binocular rivalry would occur. As a result, the switch rate of subject’s responses should be the same as the alternating rate of the grating presentations. If the interval was too short, binocular rivalry would occur, leading to slower switch rate of subject’s responses than the alternating rate of the presentations.

Based on a pilot experiment, we selected 3 candidate intervals: 250 ms, 400 ms, and 550 ms. If no binocular rivalry occurred for a subject at 250 ms or binocular rivalry occurred even at 550 ms, the interval was reduced or increased accordingly (100 ms or 700 ms). The interval test contained 3 blocks, with each corresponding to one of the 3 intervals. Each block consisted of 3 trials.

The ideal interval for each subject was determined by choosing the shortest interval at which the switch rate of responses was basically equal to the alternating rate of gratings. This was also verified by the subjective report that binocular rivalry did not occur. Figure 4c showed the result for one subject. The response ratio was calculated by dividing the switch rate of responses by the alternating rate of gratings. A response ratio of 1 means that the subject did not experience binocular rivalry at all at the given interval. A response ratio smaller than 1 indicates the existence of binocular rivalry.

For 4 subjects, the ideal interval was 400 ms, while 700 ms for 3 subjects and 550 ms for the rest of all subjects. In the formal experiment 3, the dichoptic movie was presented in alternation across the 2 eyes at each subject’s ideal interval.

It should be noted that using the ideal interval one would perceive the 2 video streams alternating roughly twice per second (~500 ms interval for most subjects). Because only the regular movie was logic and synchronized with the sound, one would be able to keep watching the regular movie stream while ignoring the frequent and short interruptions by the backward movie stream. We had verified this by ourselves before running experiment 3. This was also confirmed by all subjects’ subjective reports. Moreover, all subjects were able to retell the movie logic. All this evidence can demonstrate that subjects could pay attention to the regular movie during adaptation. We could not include a blob detection task in experiment 3 because the ideal interval was too short (~500 ms), making it very difficult to present a monocular blob as in experiment 1b in which the duration of a blob was 5.4 s in total.

Other stimuli and procedures were similar to those in experiment 1.

#### Experimental design

Experiment 3 consisted of 3 phases: (a) 3–7 days of practice on the binocular rivalry task, (b) interval test, and (c) 2 adaptation sessions. In each adaptation session, 5 binocular rivalry trials were first completed as a warm-up. A binocular rivalry pre-test (16 trials) was then conducted, followed by a 1-h adaption to the alternating version of dichoptic movie (Fig. 4a). During adaptation, subjects’ only task was to watch the movie and try their best to follow the logic of the regular movie that was presented to their dominant eyes (i.e. the attended eyes). At the end of adaptation, subjects completed a binocular rivalry post-test (16 trials).

## Results

### Experiment 1a

Here we focus on the 16 subjects who completed all adaptation conditions (see the Supplementary Material for the results of 22 subjects). A 2 (Eye Dominance: presenting the regular movie to the dominant or non-dominant eye, abbreviated as Dominant vs. Non-dominant) × 2 (Eye Status: attended vs. unattended eye) × 3 (Adaptation Condition: “Synchrony,” “Asynchrony,” or “No-sound”) repeated measurements analysis of variance (ANOVA) revealed a significant main effect of Eye Status (*F*(1,15) = 25.06, *P* < 0.001, η^2^ = 0.63) showing a higher duration ratio for the unattended eye than for the attended eye. This indicated that the balance between the 2 eyes was shifted towards the unattended eye after adaptation. There was also a significant main effect of Eye Dominance (*F*(1,15) = 10.19, *P* = 0.006, η^2^ = 0.41), but not Adaptation Condition (*F*(1.31,19.61) = 1.86, *P* = 0.19, η^2^ = 0.11, Greenhouse–Geisser corrected). The 2-way interactions were all nonsignificant (all *P*s > 0.1). Interestingly, the 3-way interaction was significant (*F*(2,30) = 4.37, *P* = 0.022, η^2^ = 0.23).

As shown by the simple effect analysis, when the dominant eye viewed the regular movie during adaptation, the duration ratio for the unattended eye was significantly larger in the “No-sound” condition than in the “Asynchrony” condition (Fig. 1c, *t*(15) = 2.73, *P* = 0.016, Cohen’s *d* = 0.68, 95% confidence interval (CI) = [0.01 0.08], *P*_FDR_ = 0.048, false discovery rate (FDR) correction); whereas the duration ratio for the attended eye in both the “No-sound” and “Synchrony” conditions was smaller than that in the “Asynchrony” condition (“No-sound” vs. “Asynchrony,” *t*(15) = −2.50, *P* = 0.027, *d* = 0.63, 95% CI = [−0.07, −0.004], *P*_FDR_ = 0.048; “Synchrony” vs. “Asynchrony,” *t*(15) = −2.33, *P* = 0.032, *d* = 0.58, 95% CI = [−0.05, −0.003], *P*_FDR_ = 0.048).

To further understand the 3-way interaction, we calculated an “adaptation score.” Specifically, for each Eye Dominance level (Dominant vs. Nondominant), the duration ratio for the unattended eye was divided by that for the attended eye, yielding an eye-status change. The eye-status change for the Dominant level was further divided by that for the Non-dominant level to produce an adaptation score for each adaptation condition. Thus, larger adaptation score means that ocular dominance shifts towards the unattended eye to a larger extent when the dominant eye views the regular movie than when the nondominant eye does. We found that the adaptation score in the “No-sound” condition was significantly larger than that in the “Asynchrony” condition (*t*(15) = 2.73, *P* = 0.015, *d* = 0.68, 95% CI = [0.03 0.25], *P*_FDR_ = 0.045). No significant difference was found between other paired comparisons (*P*s_FDR_ = 0.236).

### Experiment 1b

In experiment 1a, subjects were required to follow the regular movie in the attended eye. According to the eye-based attention literature (Chong et al. 2005; Zhang et al. 2012; Marx and Einhauser 2015), the attended eye should be more dominant during adaptation. To verify this, we used the logic of selective-looking (Neisser and Becklen 1975) and spatial attention work (Posner and Petersen 1990) to examine whether detection performance was better in the attended eye than in the unattended eye.

**Fig. 5:**
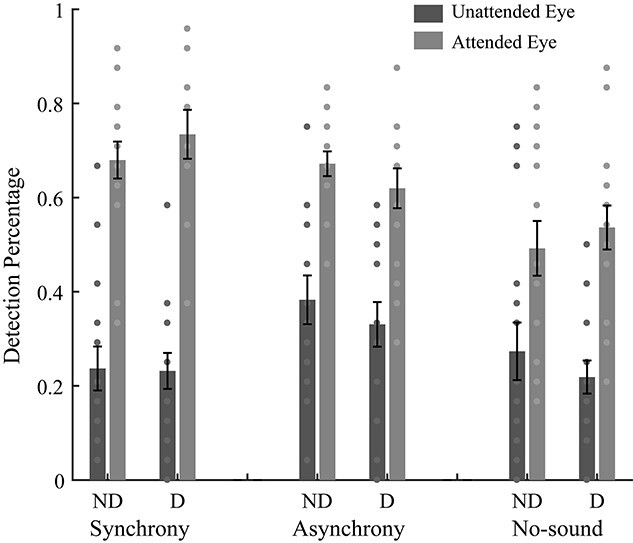
Illustration of the blob detection results for the 3 adaptation conditions of experiment 1b. The bars show the grand average detection percentages for each eye and each condition. D and ND mean presenting the regular movie to the dominant or nondominant eye, respectively. The circles show the individual data. Error bars represent standard errors of means.

For each experimental condition, the summed number of detected trials across sessions was divided by the total number of trials (24), yielding a detection percentage. A 2 (Eye Dominance: Dominant vs. Non-dominant) × 2 (Eye Status: attended vs. unattended eye) × 3 (Adaptation Condition: “Synchrony,” “Asynchrony,” or “No-sound”) repeated measurements ANOVA was performed. As expected, we found a significant main effect of Eye Status (*F*(1,15) = 134.91, *P* < 0.001, η^2^ = 0.90) showing a substantially higher detection accuracy for the attended eye (*M* = 62%) than for the unattended eye (*M* = 28%, see Fig. 5). Neither the main effect of Eye Dominance (Fig. 5, *F*(1,15) = 0.24, *P* = 0.631, η^2^ = 0.02) nor the Eye status × Eye dominance interaction (*F*(1,15) = 0.46, *P* = 0.510, η^2^ = 0.029) was significant. These results indicated that the regular movie images dominated the subject’s perception in most of the time, regardless of whether they were presented to the dominant eye or to the nondominant eye. Therefore, we believe that subjects mostly paid attention to the attended eye when watching the dichoptic movie.

In addition, we found a significant main effect of adaptation condition (Fig. 5, *F*(2,30) = 7.02, *P* = 0.003, η^2^ = 0.32) showing better performances for the “Synchrony” (*t*(15) = 2.69, *P* = 0.017, *d* = 0.67, 95% CI = [0.02 0.16], *P*_FDR_ = 0.026) and “Asynchrony” (*t*(15) = 3.37, *P* = 0.004, *d* = 0.84, 95% CI = [0.04 0.20], *P*_FDR_ = 0.013) conditions as compared to the “No-sound” condition. This suggested that the detection performances were worse when subjects watched the silent movies. Though we are not certain, this result pattern presumably reflects subject’s lower arousal level in the “No-sound” condition.

**Fig. 6:**
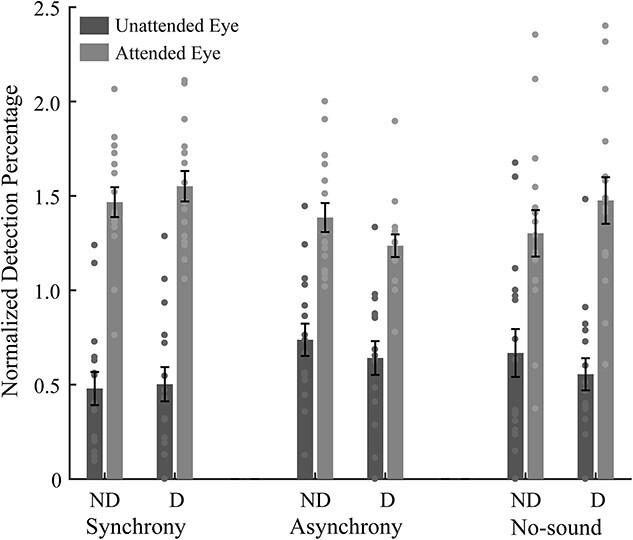
Illustration of the normalized detection percentages for the 3 adaptation conditions of experiment 1b. The bars show the grand average normalized detection percentages for each eye and each condition. D and ND mean presenting the regular movie to the dominant or nondominant eye, respectively. The circles show the individual data. Error bars represent standard errors of means.

To reduce the influences of arousal by sound, we normalized the detection percentage by dividing it by the mean detection percentage in each adaptation condition, then conducted the same repeated measurements ANOVA. We found a significant main effect of Eye Status (*F*(1,15) = 73.60, *P* < 0.001, η^2^ = 0.83, Fig. 6) and the Eye Status × Adaptation Condition interaction (*F*(2,30) = 4.66, *P* = 0.017, η^2^ = 0.24). Paired *t*-test indicated that the eye-status difference (attended-eye minus unattended-eye) of the normalized detection percentage was significantly larger in the “Synchrony” condition than in the “Asynchrony” condition (*t*(15) = 3.75, *P* = 0.002, *d* = 0.94, 95% CI = [0.17 0.62], *P*_FDR_ = 0.006). We did not find a significant difference of “Synchrony” vs. “No-sound” (*t*(15) = 1.48, *P* = 0.160, *d* = 0.37, 95% CI = [−1.07 0.59], *P*_FDR_ = 0.202) and “No-sound” vs. “Asynchrony” (*t*(15) = 1.33, *P* = 0.202, *d* = 0.33, 95% CI = [−0.09 0.41], *P*_FDR_ = 0.202). Other main effects and interactions were not significant (all *P*s > 0.283).

### Experiment 2

#### Binocular rivalry task

**Fig. 7:**
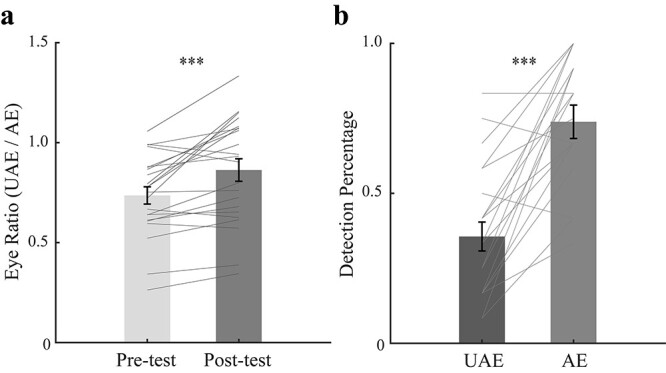
The results for (a) the binocular rivalry test and (b) the blob detection test. The bars in (a) show the grand average eye ratios (unattended eye/attended eye) in the pre- and post-tests. The bars in (b) show the grand average detection percentages for the 2 eyes. UAE means unattended eye, AE means attended eye. The gray lines show the individual data. Error bars represent standard errors of means. Asterisks represent statistically significant differences between different conditions (^***^P < 0.001).

The eye-ratio index became larger in the post-test (*M* = 0.87, SE = 0.06) than in the pre-test (*M* = 0.74, SE = 0.04; *t* (21) = 4.16, *P* < 0.001, *d* = 0.89, 95% CI = [0.06 0.19]), again suggesting that adaption shifted the perceptual ocular dominance towards the unattended eye (Fig. 7a).

#### Blob detection task

As expected, detection percentage was higher when the blob was presented to the attended eye (*M* = 0.74, SE = 0.06) than to the unattended eye (*M* = 0.36, SE = 0.05; *t* (21) = 6.49, *P* < 0.001, *d* = 1.38, 95% CI = [0.26 0.51], Fig. 7b).

#### E‌EG—test phase

A 2 (Test Phase: pre- vs. post-test) × 2 (Eye Status: attended vs. unattended eye) repeated measurements ANOVA on the average SSVEP amplitude of all EOIs revealed a significant main effect of Eye Status (*F* (1,21) = 33.00, *P* < 0.001, η^2^ = 0.61) showing higher SSVEP amplitude for the unattended-eye condition than for the attended-eye condition. No other effect was significant (*F*s < 0.23, *P*s > 0.637).

Restricting the analysis on the occipital EOIs did not show any significant effect (*F*s < 2.25, *P*s > 0.149). Accordingly, the significant main effect of Eye Status should not be explained by ocular dominance. In fact, as shown in Supplementary Fig. S4, the result was mainly contributed by the frontal EOIs.

Considering that the identical regular movie images (except for the flickering frequency) were presented to both eyes, and the SSVEP amplitude for the unattended-eye condition was higher than that for the attended-eye condition (Supplementary Fig. S4), it is unreasonable to ascribe the result in the test phase to eye-specific attention or ocular dominance. Instead, the significant main effect of Eye Status was likely due to different flickering frequencies, as neurons might be tuned to different temporal frequencies. This also explains the inclusion of a control session for normalizing the EEG data during the adaptation phase.

#### E‌EG—adaption phase

**Fig. 8:**
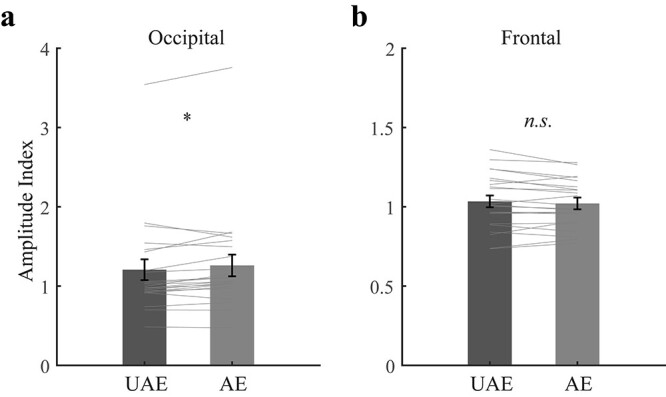
The results of the (a) occipital and (b) frontal normalized amplitude index. UAE means unattended eye, AE means attended eye. Bars show the grand average normalized amplitude indices (experimental session/control session). Gray lines show the individual data. Error bars represent standard errors of means. Asterisk represents significant difference between the unattended-eye and attended-eye conditions (^*^P < 0.05; n.s. *P* > 0.05).

To avoid the potential frequency-dependent variances, we divided the SSVEP amplitudes for the experimental session by those for the control session to obtain a normalized amplitude index for the occipital and frontal EOIs, respectively. Paired *t*-tests showed that the amplitude index was larger for the attended eye than for the unattended eye in the occipital EOIs (*t* (21) = 2.50, *P* = 0.021, *d* = 0.53, 95% CI = [0.01 0.10], Fig. 8) but not in the frontal EOIs (*t* (21) = −1.30, *P* = 0.207, *d* = 0.28, 95% CI = [−0.03 0.01]), suggesting that the regular movie images produced significantly greater visual responses than the backward ones.

We then explored if there was any functional connectivity between the frontal and occipital EOIs. The adaptation phase included three 20-min blocks. Within each block, the low-level inputs for the 2 eyes were identical (except for the image sequence). Thus, for each of the 4 conditions, i.e. 2 (tagged frequency: attended eye vs. unattended eye) × 2 (EOIs: occipital vs. frontal), we computed the SSVEP amplitude for each block in each subject, yielding 66 data points which were then subjected to the subsequent analysis. All the data followed normal distribution as evaluated by Kolmogorov–Smirnov tests (all *P*s > 0.122).

For the experimental session, there was a positive correlation of the SSVEP amplitude between the attended- and unattended-eye conditions within the same EOIs (frontal, *r* = 0.96, *P*_FDR_ < 0.001; occipital, *r* = 0.98, *P*_FDR_ < 0.001). Moreover, for all possible combinations, the frontal SSVEP amplitudes positively correlated with the occipital SSVEP amplitudes (*r*s > 0.38, *P*s_FDR_ < 0.002). On the premise of linear relationship between these data (Korn 1984), we then performed a partial correlation on the 4 conditions to regress out the contributions from other conditions, which evaluated a direct connectivity between 2 conditions (Dawson et al. 2016).

**Fig. 9:**
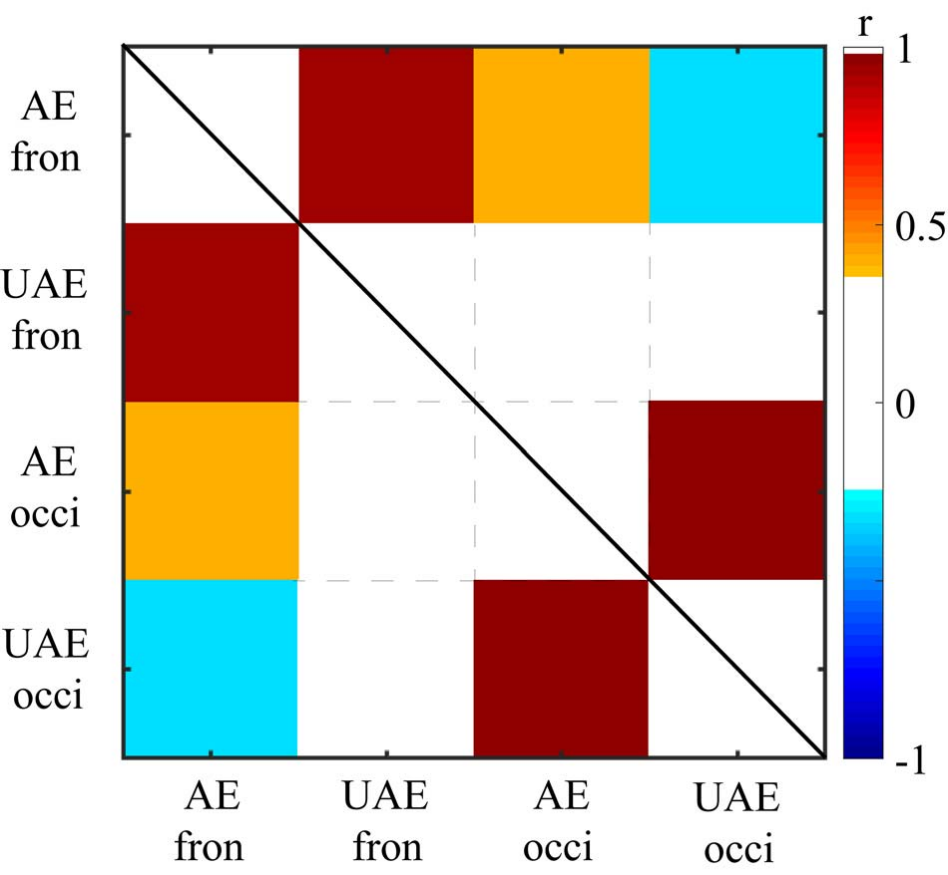
The results of partial correlation analysis of frontal and occipital activation in the attended- and unattended-eye conditions. Colors represent correlation coefficients that reached statistical significance (*P*_FDR_ < 0.05). “AE fron” means the frontal site in the attended-eye conditions; “UAE fron” means the frontal site in the unattended-eye conditions; “AE occi” means the occipital site in the attended-eye conditions; “UAE occi” means the occipital site in the unattended-eye conditions.

The results showed a local connectivity between the attended- and unattended-eye conditions within the frontal (*r* = 0.94, *P*_FDR_ < 0.001) and occipital sites (*r* = 0.98, *P*_FDR_ < 0.001). The local connectivity within the same electrode could be caused by some trivial artifacts (e.g. fluctuations in impedance), which is not of our research interest. More important, we found a fronto-occipital connectivity for the attended-eye frequency (*r* = 0.38, *P* = 0.002, *P*_FDR_ = 0.004, Fig. 9) and a negative connectivity between the frontal site for the attended-eye frequency and the occipital site for the unattended-eye frequency (*r* = −0.31, *P* = 0.012, *P*_FDR_ = 0.018). These results indicated that the frontal site played a key role in coordinating the occipital signals elicited by both the regular and backward movie images, which will be discussed in detail later.

To exclude the possibility that the flickering frequencies caused the fronto-occipital connectivity pattern, we did the same analysis for the control session. Despite significant correlations between the attended- and unattended-eye conditions within the same EOIs (frontal, *r* = 0.95, *P*_FDR_ < 0.001; occipital, *r* = 0.99, *P*_FDR_ < 0.001), no significant correlation was found between the frontal and occipital EOIs (*P*s_FDR_ > 0.977), thus ruling out the account of flickering frequency.

**Fig. 10:**
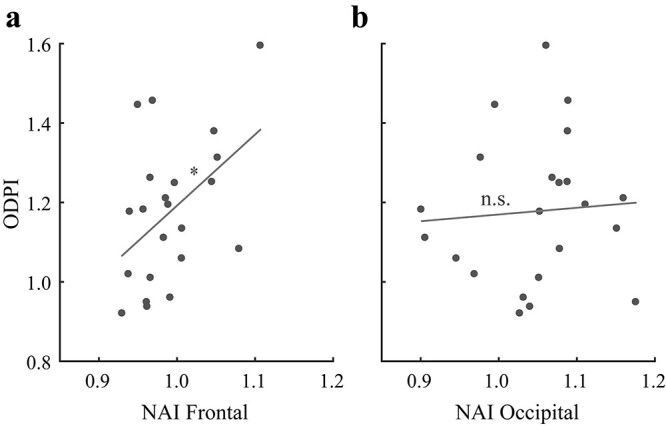
Illustration of the correlations between the ODPI and NAI in the (a) frontal and (b) occipital sites during adaptation. Asterisks represent significant correlations between the 2 conditions (^*^P < 0.05; n.s. *P* > 0.05).

Next, we investigated the relationship between perceptual ocular dominance plasticity and attention-related neural activity during adaptation. We first calculated an ocular-dominance-plasticity index (ODPI) by dividing the eye-ratio index for the post-test by that for the pre-test. An ODPI greater than 1 means a shift of ocular dominance towards the unattended eye following adaptation, with larger value indicating larger shift. To calculate a neural-activity index (NAI) related to selective attention, for each region of EOIs we divided the normalized amplitude index for the attended-eye frequency by that for the unattended-eye frequency. Although the movie images in either eye could drive SSVEP signals, selective attention to the regular movie is expected to strengthen (weaken) the signals elicited by the regular (backward) movie images. Thus, larger NAI would mean stronger attentional effect at the corresponding cortical site. Interestingly, we found a significant correlation between the ODPI with the frontal NAI (*r* = 0.46, *P* = 0.030, Fig. 10), but not with the occipital NAI (*r* = 0.07, *P* = 0.760). Because the frontal activity should be more related to higher cognitive functions (e.g. attention) than lower visual processing, these results further support the account that selective attention participates in reshaping ocular dominance in the present adaptation paradigm.

### Experiment 3

Using the blob detection task in both experiments, we verified that attention was allocated more to the eye that viewed the regular movie. This was further supported by a moderate bias of the SSVEP amplitude index in favor of the attended eye during adaptation. Importantly, both experiments indicated that prolonged attention to one eye could shift the perceptual ocular dominance towards the unattended eye. Moreover, the magnitude of this behavioral effect positively correlated with the attention-related neural activity (i.e. the advantage of neural response to the attended-eye over unattended-eye signals) particularly in the frontal site, an area often related to high-level cognitive functions.

One thing unclear is to what extent this attention-driven ocular dominance plasticity is related to binocular rivalry mechanisms, considering that dissimilar movie images in the two eyes always rivaled. Therefore, experiment 3 adopted video-alternating stimuli presented monocularly to reduce interocular competition (Fig. 4a), with the regular movie always presented to the dominant eye and the backward movie to the opposite eye.

**Fig. 11:**
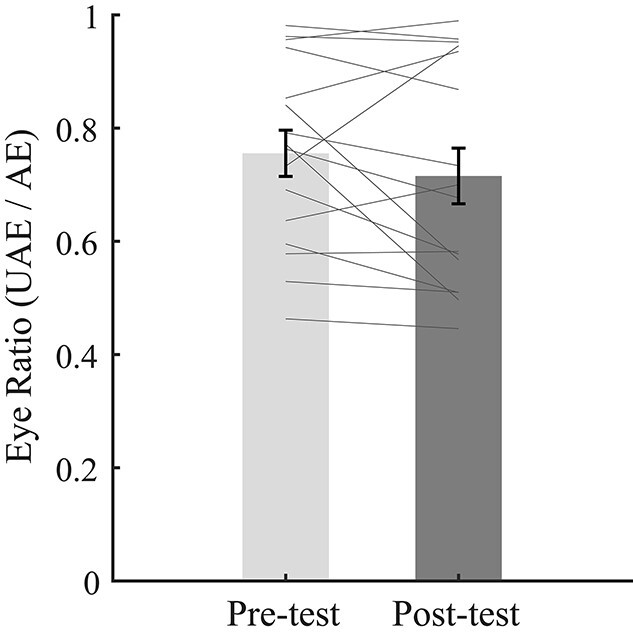
The results for the binocular rivalry task in experiment 3. The bars show the grand average eye ratios (unattended eye/attended eye) of the pre-test and post-test. The gray lines show the individual data. Error bars represent standard errors of means.

Paired *t*-test did not show a significant difference of the eye-ratio index between the pre-test (*M* = 0.76, SE = 0.04) and post-test (*M* = 0.72, SE = 0.05; *t* (15) = 1.32, *P* = 0.206, *d* = 0.33, 95% CI = [−0.02 0.10] Fig. 11).

## Discussion

The present study introduces a “dichoptic-backward-movie” adaptation paradigm. Subject’s one eye viewed the regular movie, with the opposite eye viewing the backward movie. Experimental instructions and subjects’ motivations required allocating attention to the eye that viewed the regular movie, which was further confirmed by the superior detection performances and SSVEP amplitudes for the attended eye. Importantly, 1 h of adaptation to this dichoptic movie biased the eye dominance in favor of the unattended eye.

There were crucial clues pointing to a critical role of top-down attention in driving the aftereffect (i.e. shift of perceptual ocular dominance). Firstly, the aftereffect in experiment 1a was the strongest in the “No-sound” condition (when the dominant eye viewed the regular movie). Because of lacking auditory cues, subjects in this condition must rely solely on visual inputs to identify the more logic episode and more predictable sequence among the 2 video streams and to maintain attention on the regular video stream. Considering the correspondence between attentional effort and aftereffect, top-down attention is thought to play a key role in producing the aftereffect.

Secondly, experiment 2 disclosed an interesting correlation in the frontal site between the size of behavioral aftereffect and the biased neural activity in favor of the attended eye during adaptation. This finding highlights the possible contribution of frontal attentional rather than pure visual mechanisms to the aftereffect. One may question that the frontal activity might not directly reflect the attentional effect. Here we first exclude the account of eye movement, since eye movement-related activations should not be revealed through frequency tagging. Actually, any brain activity, as long as not oscillating at the flickering frequencies as the movie images, would be missed out through frequency tagging. For instance, our subjects certainly paid attention to the audio stimuli during adaptation, yet no electrode at the temporal site showed a reliable response. Given this common sense, we admit that frequency tagging cannot exhaustively detect all attention-related brain activities, simply because not every attention-related neural response oscillates like the visual responses elicited by the movie images.

Then what did the frequency-tagged frontal response likely reflect? An inspection of the frontal EOIs suggests that the frontal activity was mainly located at the frontal pole and medial frontal cortex. These brain areas have been proposed to be involved in humor processing and appreciation (Jääskeläinen et al. 2016; Iidaka 2017), semantic processing and sustained attention to dialogue (Xie et al. 2018; Leminen et al. 2020). Our movie was a Chinese TV comedy series called “iPartment.” Understanding the logic of comedy requires holding and reinterpreting information in light of new information several seconds later and thus relies on the activity of areas such as frontal pole that exhibits longer temporal receptive window (Jääskeläinen et al. 2016). On the other hand, understanding the logic of comedy also requires focusing attention on the regular movie images and avoiding the interferences from the backward movie images. From this perspective, the frequency-tagged response at the frontal site should be somehow related to the attentional effect, even if it was not the source of attentional signals.

Interestingly, in the adaptation phase, we found a positive fronto-occipital connectivity for the attended-eye frequency and a negative connectivity between the frontal response for the attended-eye frequency and the occipital response for the unattended-eye frequency. However, in the control session, no significant correlation was found between the frontal and occipital EOIs, excluding any account of flickering frequency or trivial artifacts for the connectivity results in the adaptation session. Presumably, the frontal areas were responsible for analyzing the comedy logic based on the visual inputs; and the video information temporarily held in the frontal areas was also likely frequency-tagged (since the visual inputs were frequency-tagged). However, useful input signals were always from the attended eye. Therefore, the positive fronto-occipital connectivity might reflect an attentional enhancement coordinating the frontal response for the attended-eye frequency with the input signals from the attended eye, whereas the negative fronto-occipital connectivity might reflect a suppression on the input signals from the unattended eye. These connectivity findings together with the behavior–SSVEP correlation result strongly suggest that the prolonged eye-specific attention was responsible for the aftereffect.

Nevertheless, as shown by experiment 3, attention cannot work alone to bias perceptual ocular dominance and probably has to co-work with binocular rivalry mechanisms, since the aftereffect disappeared when interocular competition was substantially reduced. Therefore, we tend to explain our findings in the framework of the ocular-opponency-neuron model of binocular rivalry (Said and Heeger 2013). According to the model, ocular opponency neurons compute the difference in the signals between the 2 eyes (Said and Heeger 2013; Katyal et al. 2016, 2018). The opponency neurons receive excitation from one eye and inhibition from the other eye. Accordingly, they are active only when the excitatory signals outweigh the inhibitory signals. Meanwhile, when active, the opponency neurons also inhibit the monocular neurons from which they receive inhibition.

For simplicity, suppose the right eye was the attended eye. Then in experiment 3, the R-L opponency neurons would activate whenever the regular movie was presented to the right eye and blank field to the left eye. Whenever the contrary occurred, the L–R opponency neurons would activate. Since the summed duration was equal for either type of interval, the L–R and R–L opponency neurons would adapt to the same extent. Thus, the relative mutual inhibition of the 2 eyes’ inputs would be similar in the post- and pre-tests.

In experiments 1 and 2, however, dissimilar movie images were simultaneously presented to the 2 eyes, causing interocular competition. Attending to the regular movie strengthened (suppressed) the neural responses of monocular neurons for the right (left) eye, allowing the regular movie to be perceived for most of the time. Therefore, the R–L opponency neurons adapted to a larger extent than the L–R opponency neurons. This would cause the unattended eye (i.e. left eye) to receive less inhibition from the R–L opponency neurons in the binocular rivalry post-test, thus shifting the ocular dominance towards the unattended eye.

Moreover, the absence of effect in the EEG post-test further supports our account. Unlike the adaptation phase and behavioral pre- and post-tests, the binocularly compatible (or fused) stimuli were presented to the 2 eyes in the EEG pre- and post-tests. Processing of such binocularly fused stimuli were least affected by opponency neurons, based on the definition of this type of neurons. Therefore, the adaptation effect of the opponency neurons in experiment 2 could not be manifested by using binocularly fused testing stimuli. As a direct comparison, our previous monocular deprivation study also adopted binocularly fused flickering stimuli in the EEG pre- and post-tests (Lyu et al. 2020). However, that study successfully discloses a shift of neural ocular dominance in favor of the deprived eye, suggesting that monocular deprivation can affect mechanisms that are active when information from the 2 eyes does not conflict. Yet a null effect found in the present EEG post-test shows one fundamental way in which the effects of attention in ocular dominance plasticity differ from the effects of monocular deprivation (Lunghi et al. 2015; Lyu et al. 2020).

Intuitively, larger shift of ocular dominance should be associated with more imbalanced perceptions of each eye’s inputs during adaptation, which may be associated with more imbalanced neural responses between the 2 eyes. Although we found stronger SSVEP amplitude index for the attended eye than for the unattended eye, unfortunately, the ODPI did not significantly correlate with the occipital NAI. Instead, it positively correlated with the frontal NAI. These results imply that the frontal attentional system somehow modulated the AE-UAE ocular opponency neurons (AE denotes attended eye; UAE denotes unattended eye). Accordingly, even if there was no large imbalance of monocular visual responses during adaptation, sufficiently strong attentional enhancement on the AE-UAE opponency neurons could still produce a large aftereffect. This bold speculation agrees with the finding in experiment 1 that the high-demanding “No-Sound” condition did not show the largest interocular difference of detection performance (an indirect hallmark of imbalanced monocular visual responses), yet it generated the largest aftereffect.

The present study opens the route to explain at least part of short-term ocular dominance plasticity with the ocular-opponency-neuron model (Said and Heeger 2013). Our adaptation paradigm differs from typical monocular deprivation, since no sensory information in either eye is blocked. Even so, the model is promising for explaining various monocular deprivation effects whenever interocular competition exists during adaptation, which may be a useful complement to the homeostatic compensation theory that has been prevalently used to explain the effects of short-term monocular deprivation (Lunghi et al. 2013, 2015; Bai et al. 2017; Lyu et al. 2020).

## Author contributions

MB developed the study concept. SF, LL, and JZ performed the research. SF, LL, and MB designed the research, analyzed the data, and wrote the paper. All authors approved the final version of the manuscript for submission.

## Funding

This research was supported by the Ministry of Science and Technology of China (2021ZD0203800) and the National Natural Science Foundation of China (31871104 and 31830037).

*Conflict of interest statement*. The authors declared no conflicts of interest.

## Supplementary Material

Supplemental_Material_bhac116
