# Supplementary material for: The role of eye-specific attention in ocular dominance plasticity
Source: Cereb Cortex. 2022 Mar 24;33(4):983–96. doi: 10.1093/cercor/bhac116 (PMC9930618; doi:10.1093/cercor/bhac116)
Supplement: Supplemental_Material_bhac116 [file supplemental_material_bhac116.docx]

**The role of eye-specific attention in ocular dominance plasticity**

Fangxing Song, Lili Lyu, Jiaxu Zhao, Min Bao

**Supplemental Results for Experiment 1a**

Table 1. Grand average duration ratios (mean [95% CI]) for each condition.

| Condition (number of subjects) | Unattended eye | | Attended eye | |
| --- | --- | --- | --- | --- |
|  | Non-dominant | Dominant | Non-dominant | Dominant |
| Synchrony (22) | 1.02 [1.00, 1.05] | 1.05 [1.02, 1.08] | 0.97 [0.95, 1.00] | 0.96 [0.94, 0.98] |
| Synchrony (16) | 1.03 [1.00, 1.06] | 1.06 [1.03, 1.09] | 0.97 [0.94, 1.00] | 0.95 [0.93, 0.98] |
| Asynchrony (16) | 1.03 [1.00, 1.05] | 1.03 [0.99, 1.07] | 0.97 [0.94, 1.00] | 0.98 [0.95, 1.01] |
| No-sound (16) | 1.01 [0.97, 1.04] | 1.07 [1.03, 1.12] | 1.00 [0.95, 1.04] | 0.94 [0.92, 0.97] |

Table 1 listed the grand average duration ratios for the three adaptation conditions. A 2×2 repeated measurements ANOVA with Eye Dominance (presenting the regular movie to the dominant vs. non-dominant eye, abbreviated as Dominant vs. Non-dominant) and Eye Status (the attended vs. unattended eye) as factors was used to statistically analyze the results (Fig. S1). As predicted, we found a significant main effect of Eye Status (*F*(1,21) = 19.81, *p* < .001, η^2^ = .49) showing a higher duration ratio for the unattended eye than for the attended eye (see Table 1 for more details). This indicated that the balance between the two eyes was shifted towards the unattended eye after adaptation. The main effect of Eye Dominance was also significant (*F*(1,21) = 9.03, *p* = .007, η^2^ = .30). However, the two-way interaction was non-significant (*F*(1,21) = 1.35, *p* = .258, η^2^ = .06).


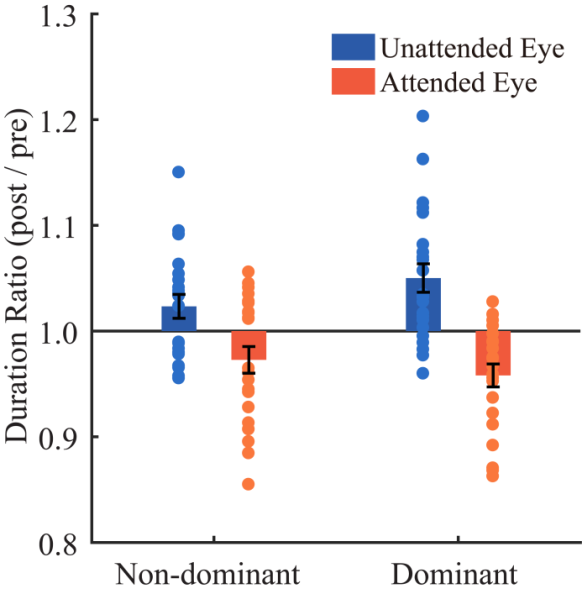


**Fig. S1.** Illustration of the mean duration ratios for the “Synchrony” adaptation condition (N = 22) in Experiment 1a. Error bars represent standard errors of means.

For the 16 subjects’ data in the main text, we also tried the analysis with the eye-ratio index (see the definition in Experiment 2). We also used this index in the present Experiment 2 and our previous work ([Bai et al. 2017](#_ENREF_1); [Lyu et al. 2020](#_ENREF_4)).


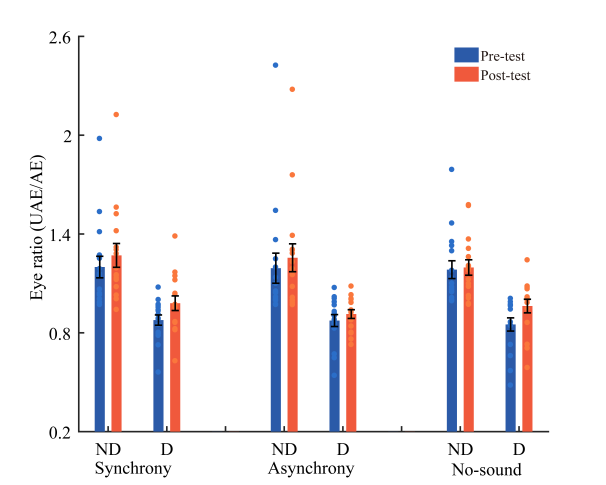


**Fig. S2.** The mean eye ratios for the three adaptation conditions in Experiment 1a. The circles show the individual data. Error bars represent standard errors of means.

Specifically, we conducted a 2 (Eye Dominance: viewing the regular movie with the dominant or non-dominant eye) × 2 (Test Phase: pre vs. post) × 3 (Adaptation Condition: “Synchrony”, “Asynchrony”, or “No-sound”) repeated measurements ANOVA. There was a significant main effect of Test Phase (*F*(1,15) = 18.46, *p* = 0.0006) showing increased eye-ratio index following adaptation, an indication of ocular dominance shift towards the unattended eye. We also found a significant main effect of Eye Dominance (*F*(1,15) = 15.10, *p* = 0.0015), but not Adaptation Condition (*F*(2,30) = 0.57, *p* = 0.57). The two-way interactions were all non-significant (all *p*s > 0.39). There was also a significant three-way interaction (*F*(2,30) = 4.08, *p* = 0.027).

To further understand the three-way interaction, we also calculated an *adaptation score*. Specifically, for each Eye Dominance level (Dominant vs. Non-dominant), the eye-ratio index in the post-test was divided by that in the pre-test, yielding an eye-status change. The eye-status change for the Dominant level was further divided by that for the Non-dominant level to produce an adaptation score for each adaptation condition. Thus, larger adaptation score means that ocular dominance shifts towards the unattended eye to a larger extent when the dominant eye views the regular movie than when the non-dominant eye does. We found that the adaptation score in the “No-sound” condition was significantly larger than that in the “Asynchrony” condition (*t*(15) =2.73, *p* = .016, *d* = 0.68, 95% CI = [0.03 0.25], *p*_FDR_ = .047). No significant difference was found between other paired comparisons (*p*s_FDR_ = 0.236).

Therefore, the ANOVA and adaptation score results for the eye-ratio index reported here generally resembled those for the duration-ratio index reported in the main text.

**Supplemental Methods for Experiment 2**

***Stimuli and Procedure***


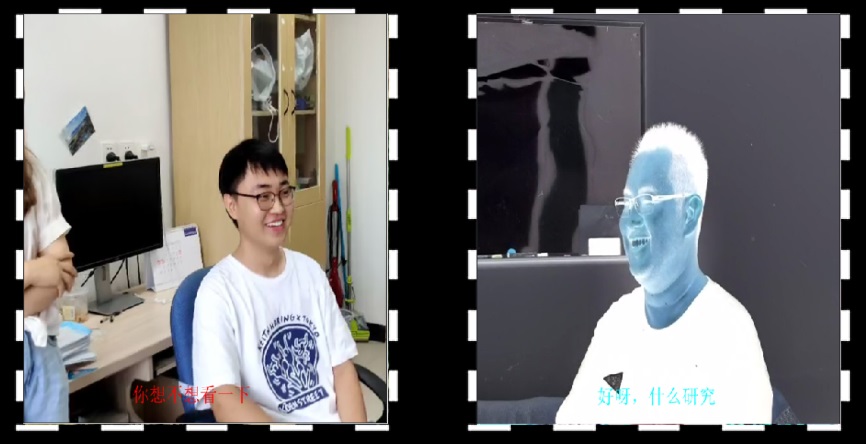


**Fig. S3.** Illustration of the contrast-reversed flickering

***Data Analysis***

***Selection of electrodes of interest (EOIs).*** To focus on the electrodes with sufficiently strong responses, for each electrode we compared the average SSVEP amplitude for both eyes in the pre- and post-tests with the grand mean amplitude across all the electrodes and subjects by using a one-sample *t*-test ([Huang et al. 2018](#_ENREF_3); [Dong et al. 2020](#_ENREF_2); [Lyu *et al.* 2020](#_ENREF_4)). Only those electrodes whose SSVEP amplitude was significantly greater than the grand mean were selected as EOIs for further analysis.

In the test phase, the electrodes (FP1 FPZ FP2 FZ F8 CB1 CB2) were selected as the EOIs. Then the SSVEP amplitude was averaged across the EOIs for statistical comparisons (Fig. 3). In the adaption phase, we used the same method for electrode selection as in the test phase. We first selected electrodes for the experimental session. A total of ten electrodes were selected as the EOIs, which were divided into frontal electrodes (FP1 FPZ FP2 AF3 AF4 FZ F8) and occipital electrodes (POZ CB1 CB2) according to the region which the EOI belonged to (Fig. 3). We then selected electrodes for the control session. The electrodes (FP1 FPZ FP2 AF4 F7 FZ F4 F8 T8 TP7 POZ CB2) were selected as the EOIs. Since the EOIs for the two sessions differed slightly, and the data of the control session was mainly used in a normalization analysis for those of the experimental session, we eventually used the EOIs for the experimental session to analyze the data in both sessions.

**Supplemental Results for Experiment 2**

***Additional results of EEG – Test phase***


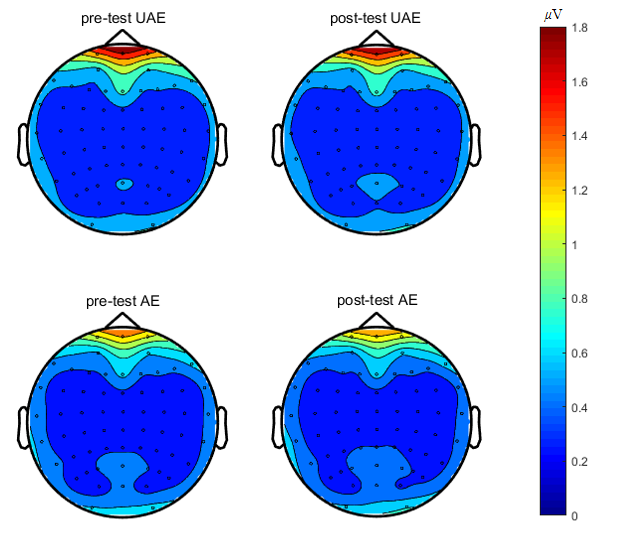


**Fig. S4.** Average topography for the test phase. Here UAE represents the unattended-eye condition, while AE represents the attended-eye condition.

***Additional results of EEG – Adaptation phase***

As shown in Fig. 8a, the amplitude index of one subject was much larger than those of other subjects. Therefore, we tried performing the paired t-test after excluding this subject’s data. Again, the amplitude index was larger for the attended eye than for the unattended eye in the occipital EOIs (*t* (20) = 2.18, *p* = 0.041, *d* = 0.48, 95% CI = [0.002 0.091]).

**References**

Bai J, Dong X, He S, Bao M. 2017. Monocular deprivation of Fourier phase information boosts the deprived eye's dominance during interocular competition but not interocular phase combination. Neuroscience. 352:122-130.

Dong X, Du X, Bao M. 2020. Repeated Contrast Adaptation Does Not Cause Habituation of the Adapter. Front Hum Neurosci. 14:589634.

Huang Q, Jia J, Han Q, Luo H. 2018. Fast-backward replay of sequentially memorized items in humans. eLife. 7.

Lyu L, He S, Jiang Y, Engel SA, Bao M. 2020. Natural-scene-based Steady-state Visual Evoked Potentials Reveal Effects of Short-term Monocular Deprivation. Neuroscience. 435:10-21.
